# Paper Plates

**Published:** 1881-08

**Authors:** 


### PAPER PLATES.

The latest application of paper is the adoption of paper plates by some of the great restaurants and cafés in Berlin. The innovation was first introduced during the summer of last year by the adventurous landlord of a much-frequented open-air restaurant. Every customer who ordered bread and butter, rolls, cakes, buns or similar articles, had them served to him upon a little paper plate, made of light papier maché, adorned with a pretty border of relief, an having at the first glance a great similarity to porcelain. Guests, waiters and host were all pleased with the novelty; it saved the waiters many a deduction from their wages on account of breakages, which the deftest and cleverest can scarcely avoid when he handles hundreds of pieces of crockery during a single afternoon and evening. The paper plates were so cheap that the landlord did not care to assert his ownership over them, and his customers were allowed to carry them away, like the petty serviettes of thin paper used in so many restaurants in Holland. There was also a considerable saving of the time lost, and the chance of accident incurred in the cleansing of earthenware pottery. The success of the experiment has been so marked that the new species of plates is likely to be introduced into a great number of restaurants.

Broke the Car-String.—As a train was approaching Cleveland it parted in the middle, and the bell-rope snapped off like a thread, the end of it striking an old lady on her bonnet.

"What is the matter?" she exclaimed.

"Oh, the train's broke in two," replied a gentleman who sat in the next seat.

"I should say so," the old lady said, looking at the broken bell cord. "Did they s'pose a trifling little string like that would hold the train together?"

